# Vancomycin Tolerant, Methicillin-Resistant *Staphylococcus aureus* Reveals the Effects of Vancomycin on Cell Wall Thickening

**DOI:** 10.1371/journal.pone.0118791

**Published:** 2015-03-20

**Authors:** Vicenta Cázares-Domínguez, Ariadnna Cruz-Córdova, Sara A. Ochoa, Gerardo Escalona, José Arellano-Galindo, Alejandra Rodríguez-Leviz, Rigoberto Hernández-Castro, Edgar O. López-Villegas, Juan Xicohtencatl-Cortes

**Affiliations:** 1 Laboratorio de Investigación en Bacteriología Intestinal, Hospital Infantil de México Federico Gómez, Dr. Márquez 162, Col. Doctores, Delegación Cuauhtémoc, México D.F., México; 2 Departamento de Infectología, Hospital Infantil de México Federico Gómez, Dr. Márquez 162, Col. Doctores, Delegación Cuauhtémoc, México D.F., México; 3 Laboratorio de Patología, Hospital Infantil de México Federico Gómez, Dr. Márquez 162, Col. Doctores, Delegación Cuauhtémoc, México D.F., México; 4 Departamento de Ecología de Agentes Patógenos. Hospital General “Dr. Manuel Gea González”, Delegación Tlalpan, México D.F., México; 5 Laboratorio Central de Microscopia. Escuela Nacional de Ciencias Biológicas, Instituto Politécnico Nacional. Prol. De Carpio y Plan de Ayala S/N Col. Santo Tomás, Delegación Miguel Hidalgo, México D.F., México; Columbia University, UNITED STATES

## Abstract

Methicillin-resistant *Staphylococcus aureus* (MRSA) is an important opportunistic pathogen that causes both healthcare- and community-acquired infections. An increase in the incidence of these infections may lead to a substantial change in the rate of vancomycin usage. Incidence of reduced susceptibility to vancomycin has been increasing worldwide for the last few years, conferring different levels of resistance to vancomycin as well as producing changes in the cell wall structure. The aim of the present study was to determine the effect of vancomycin on cell wall thickening in clinical isolates of vancomycin-tolerant (VT) MRSA obtained from pediatric patients. From a collection of 100 MRSA clinical isolates from pediatric patients, 12% (12/100) were characterized as VT-MRSA, and from them, 41.66% (5/12) exhibited the heterogeneous vancomycin-intermediate *S*. *aureus* (hVISA) phenotype. Multiplex-PCR assays revealed 66.66% (8/12), 25% (3/12), and 8.33% (1/12) of the VT-MRSA isolates were associated with *agr* group II, I, and III polymorphisms, respectively; the *II-mec* gene was amplified from 83.3% (10/12) of the isolates, and the *mec*IVa gene was amplified from 16.66% (2/12) of the isolates. Pulsed field electrophoresis (PFGE) fingerprint analysis showed 62% similarity among the VT-MRSA isolates. Thin transverse sections analyzed by transmission electron microscopy (TEM) revealed an average increase of 24 nm (105.55%) in the cell wall thickness of VT-MRSA compared with untreated VT-MRSA isolates. In summary, these data revealed that the thickened cell walls of VT-MRSA clinical isolates with *agr* type II and *SCCmec* group II polymorphisms are associated with an adaptive resistance to vancomycin.

## Introduction

Methicillin-resistant *Staphylococcus aureus* (MRSA) is an important opportunistic pathogen associated with nosocomial and community-acquired invasive infections [1–4]. More than 90% of the MRSA isolates are resistant to all available penicillins and other β-lactam antimicrobial drugs, limiting the therapeutic options available to treat serious infections [5]. Vancomycin is the primary drug of choice for the treatment of MRSA infections; however, the excessive use of this antibiotic has led to the emergence of new *S*. *aureus* strains, such as vancomycin-intermediate *S*. *aureus* (VISA) and heterogeneous-VISA (hVISA). MRSA strains also develop vancomycin tolerance, commonly defined as a minimum bactericidal concentration (MBC)/minimum inhibitory concentration (MIC) ratio of ≥ 32. The infections caused by vancomycin-tolerant MRSA strains (VT-MRSA) are more difficult to treat, particularly when they are associated with endocarditis, meningitis, osteomyelitis, and infections in immunocompromised patients [6–9].

Failures in clinical treatment with vancomycin have been associated with strains having vancomycin susceptibility within the intermediate MIC range (VISA), and two mixed susceptibility subpopulations (hVISA) have also been described. Infections caused by hVISA strains are poorly understood, reflecting the difficulty of detecting low frequency subpopulations resistant to vancomycin [10–14]. Therefore, the population analysis profile (PAP) method has been used for the identification of hVISA strains [15, 16]. Recent reports from many countries have suggested that hVISA strains are responsible for failures in vancomycin therapy and that patients administered vancomycin for prolonged periods of time are potential sources of VISA subclones [17–19].

The accessory gene regulatory (*agr*) locus is a quorum-sensing operon that coordinates the expression of several housekeeping genes and genes encoding secreted and cell-associated virulence factors [20, 21]. In addition, four major *S*. *aureus agr* groups (I, II, III, and IV) associated with distinct clinical manifestations have been described [21–23]. Many VISA strains are highly enriched for the *agr* group II polymorphism [24, 25]. MRSA strains are hallmarked by the presence of a 2.1-kb *mecA* gene encoding penicillin-binding protein 2a (78 kDa) with a reduced affinity for β-lactams [26, 27]. Currently, eight major SCC*mec* types have been associated with the *mec* gene complex, and they are divided into class A (SCC*mec* types II, III, and VIII), class B (SCC*mec* types I, IV, and VI), and class C (SCC*mec* types V and VII) [28, 29].

Cell wall synthesis in VISA strains affected by metabolic changes influences the cell wall thickening mechanism [8, 30, 31]. Interestingly, cell wall thickening in VT-MRSA clinical isolates when administered with gradually increasing doses of vancomycin has not yet been described; however, both hVISA Mu3 and VISA Mu50 have been shown to use cell wall thickening as a vancomycin tolerance mechanism, suggesting that cell wall thickening is requisite for vancomycin-resistant strains. Therefore, the aim of the present study was to evaluate cell wall thickening in VT-MRSA clinical isolates from pediatric patients as a direct effect of vancomycin stimulation and examine the interrelationships among the susceptibility profiles, *agr*-group polymorphisms, SCC*mec* types, and genetic relatedness.

## Material and Methods

### Ethics statement

The study was reviewed and approved by the Research Committee (Dr. Onofre Muñoz Hernández), Ethics Committee (Dra. Amparo Faure Fontenla), and Biosecurity Committee (Dra. Herlinda Vera Hermosillo) of Hospital Infantil de México Federico Gómez (HIMFG), with permit number HIM/2010/016. The Central Laboratory from HIMFG provided the MRSA isolates for this study. The anonymous clinical information presented in this manuscript prior to analysis was obtained from the patient’s medical record, considering the diagnosis, and sample type. According to the institutional ethical, biosecurity and investigation evaluation an informed consent is not required.

### Clinical isolates of *S*. *aureus*

A collection of 100 MRSA clinical isolates from pediatric patients (one isolate per patient) kept in the Central Laboratory at the HIMFG was analyzed in this study. The patients’ medical records were reviewed to understand the clinical relevance, sample type and diagnosis. The clinical *S*. *aureus* isolates were confirmed in the laboratory using conventional methods, such as colony morphology, Gram staining, catalase activity, human plasma coagulase production, mannitol fermentation, and growth on broth heart infusion agar (BHI; Dibco, México DF, México) supplemented with 15% NaCl. In addition, methicillin resistance in all clinical *S*. *aureus* isolates was confirmed through the Kirby-Bauer method using oxacillin (Sigma-Aldrich; MO, USA) at the Clinical and Laboratory Standards Institute (CLSI) 2013 [32].

### Determination of vancomycin MIC and MBC

The vancomycin susceptibility profiles of MRSA isolates were determined using the MIC technique via the microdilution method in Mueller-Hinton broth (MHB; Becton Dickinson, México DF, México), according to the CLSI 2013 [32]. Several concentrations (128–0.50 μg/mL) of vancomycin were prepared in MHB, and 100 μL of antibiotic sample was loaded into each well of a microplate. For each dilution, 100 μl of a bacterial suspension (1.5x10^5^ bacteria/mL) was inoculated and grown at 37°C under a CO_2_ atmosphere for 20 h. The MIC values for each MRSA isolate were calculated when the bacterial colonies were completely inhibited at the lowest concentration after incubation for 20 h. To determine the MBC, 10 μL of the bacterial suspension from the MIC microplate of the well in which bacterial inhibition occurred, was spread onto blood agar, incubated for 24 h, and analyzed to determine the number of colony-forming units. *S*. *aureus* strain ATCC 29213 was used as a control. The data were interpreted according to the guidelines of the CLSI 2013 [32].

### Identification of clinical isolates of vancomycin-tolerant *S*. *aureus*

The vancomycin tolerance test was performed as previously described [7]. In addition, vancomycin tolerance is defined as a MBC/MIC ratio ≥ 32. An MIC test was performed for each clinical *S*. *aureus* isolate, followed by the MBC test. The MBC/MIC ratio test was performed as a presumptive concentration screening of the antibiotics used in the present study.

### Antimicrobial susceptibility profile for vancomycin-tolerant MRSA

The antibiotic susceptibility profiles for the VT-MRSA isolates were determined using the MIC technique via the microdilution method according to the CLSI 2013 [32], as described in the previous section. MIC tests were performed for ampicillin (Sigma-Aldrich), ceftazidime (Pfizer; México DF, México), ceftriaxone (Roche; Mexico DF, México), ciprofloxacin (Bayer; Levenkusen Westfalia, Germany), erythromycin (MP Biomedicals; Solon, OH, USA), kanamycin (Sigma-Aldrich), meropenem (Astra Zeneca; México DF, Mexico), rifampicin (MP Biomedicals), gentamicin (MP Biomedicals), and trimethoprim-sulfamethoxazole (Sigma-Aldrich).

### Population analysis profile

The VT-MRSA isolates were screened to detect the hVISA phenotype using PAP analysis as previously described [33]. From a bacterial suspension adjusted to 1x10^8^ CFU/mL were made serial dilutions (10^1^ to 10^8^), and a 25-μL inoculum of each dilution was expanded in BHI agar supplemented with vancomycin concentrations of 0 to 8 mg/L and incubated at 37°C for 48 h. The hVISA strain Mu3 and the vancomycin-susceptible *S*. *aureus* strain (VSSA) ATCC 25923 were used as controls in the present study. An isolate was defined as hVISA if the AUC of the test isolate divided by the AUC of the corresponding strain Mu3 was **≥** 0.9.

### DNA extraction

The VT-MRSA clinical isolates were cultured on blood agar for 18 to 24 h and treated with an enzymatic cocktail (2 mg/mL lysozyme, 100 μg/mL proteinase K, and 1 mg/mL lysostaphin [Sigma Aldrich; MO, USA]) for genomic DNA extraction using the commercial Wizard Genomic DNA Purification Kit (Promega Corporation, Madison, WI, USA).

### *SCCmec* typing using multiplex PCR

To characterize the SCC*mec* genes (I, II, III, IVa, V, and *mecA*), six primer pairs were used (Table 1). Briefly, 100 ng of pure DNA was added to a reaction mixture (Promega Corporation, Madison, WI, USA) containing 2.5 mM MgCl_2_, 0.2 mM of each deoxynucleoside triphosphate (dATP, dGTP, dCTP, and dTTP), 1U of Taq DNA polymerase, varying concentrations of the different oligos and nuclease-free water. The genes were amplified under the following conditions: an initial denaturation step at 94°C for 5 min; 10 cycles of denaturation at 94°C for 45 sec, annealing at 65°C for 45 sec, and extension at 72°C for 1.5 min; 25 cycles of denaturation at 94°C for 45 sec, annealing at 65°C for 45 sec, and annealing at 55°C for 45 sec; and a final extension at 72°C for 10 min. The PCR products were separated by electrophoresis on 1.5% agarose gels and subsequently stained with 0.5 mg/mL ethidium bromide dissolved in 0.5X TBE buffer (Tris-borate-EDTA). The stained gels were visualized and analyzed under ultraviolet light. The SCC*mec* type I (*S*. *aureus* 1746), type II (*S*. *aureus* 1749), type III (*S*. *aureus* 1748), and type IVa (*S*. *aureus* USA 300 and MW2) strains were used as positive controls.

**Table 1:** List of PCR primers and amplicon sizes of the target genes.

Genes	Primers	Sequences (5´– 3´)	Amplicon size (bp)	Reference
*agr* I	*agr* I-F	ATGCACATGGTGCACATGC	441	[35]
	*agr* I-R	GTCACAAGTACTATAAGCTGCGAT		
*agr* II	*agr* II-F	ATGCACATGGTGCACATGC	575	[35]
	*agr* II-R	TATTACTAATTGAAAAGTGGCCATAGC		
*agr* III	*agr* III-F	ATGCACATGGTGCACATGC	323	[35]
	*agr* III-R	GTAATGTAATAGCTTGTAAAAAGTGGCCATAGC		
*agr* IV	*agr* IV-F	ATGCACATGGTGCACATGC	659	[35]
	*agr* IV-R	CGATAATGCCGTAATACCCG		
*SCCmec* I	*mec* I-F	GCTTTAAAGAGTGTCGTTACAGG	613	[34]
	*mec* I-R	GTTCTCTCATAGTATGACGTCC		
*SCCmec* II	*mec* II-F	CGTTGAAGATGATGAAGCG	398	[34]
	*mec* II-R	CGAAATCAATGGTTAATGGACC		
*SCCmec* III	*mec* III-F	CCATATTGTGTACGATGCG	280	[34]
	*mec* III-R	CCTTAGTTGTCGTAACAGATCG		
*SCCmec* IVa	*mec* IVa-F	GCCTTATTCGAAGAAACCG	776	[34]
	*mec* IVa-R	CTACTCTTCTGAAAAGCGTCG		
*SCCmec* V	*mec* V-F	GAACATTGTTACTTAAATGAGCG	325	[34]
	*mec* V-R	TGAAAGTTGTACCCTTGACACC		
*mecA*	*mec* 147-F	GTGAAGATATACCAAGTGATT	147	[34]
	*mec* 147-R	ATGCGCTATAGATTGAAAGGAT		

F = forward and R = reverse

### *agr* typing using multiplex PCR

Multiplex PCR assays were performed to characterize the *agr* groups using specific oligonucleotides derived from the variable region of the *agr* operon [34]. In the present study, oligonucleotides for the *agr* groups (I, II, III, and IV) were synthesized according to Gilot *et al*., [35]. The primer sequences and GenBank accession numbers are shown in Table 1. The clinical isolates of *S*. *aureus* USA300 (type I, 441 bp), *S*. *aureus* 1749 (type II, 575 bp), and *S*. *aureus* ATCC 25923 (type III, 323 bp) were used as positive controls. Briefly, multiplex assays containing 1.25 U of Taq DNA polymerase, 200 mM dNTPs, 1.5 mM MgCl_2_, 0.3 mM of each oligonucleotide, 100 ng of DNA, and nuclease-free water were prepared. The DNA amplification was performed using a Thermo Hybaid thermocycler (Hybaid PCR Sprint Thermal Cyclers) under the following thermal cycling conditions: denaturation at 94°C for 5 min, annealing at 55°C for 30 sec, and extension at 72°C for 60 sec; this procedure was performed for 26 cycles. The integrity of the amplified DNA was assessed by electrophoresis on 1.5% agarose gels and visualized after staining with 0.5 mg/mL of ethidium bromide. The length of the PCR fragments was identified using 100-bp molecular weight markers (Fermentas; Vilnius, Lithuania).

### Molecular genotyping assays

Pulsed-field gel electrophoresis (PFGE) was performed using a previously described protocol [36]. The chromosomal DNA was digested with *Sma*I and subjected to electrophoresis on 1% agarose gels (Promega; Madison, WI, USA) using the following parameters: 200 V (6 v/cm) at 14°C for 21.5 h, with an initial change of 5 sec and an end change of 40 sec. The gels were stained with 1.0 mg/mL ethidium bromide solution and visualized using a gel imaging system (ChemiDoc MP System, Biorad; México). The DNA fragment patterns generated by PFGE were analyzed using NTSY program version 2.0 with the Dice coefficient and the unweighted pair group method with an arithmetic mean (UPGMA) clustering system.

### Analysis of cell-wall thickening in VT-MRSA clinical isolates

Transmission electron microscopy (MET, JOEL model JEM 10–10) was used to morphometrically visualize cell-wall thickening in the VT-MRSA isolates as an effect of antibiotic concentration. Vancomycin-treated bacteria were grown to the exponential phase in the presence of gradual increments of 1 mg/mL antibiotics to a final concentration of 20 μg/mL. As a control, untreated bacteria were also grown to the exponential phase. The bacteria were harvested, washed, and fixed with a glutaraldehyde/formalin (2.5%/10%) solution in 0.1 M phosphate-buffered saline (PBS), pH 6.0. Subsequently, the bacteria were post-fixed with osmium tetroxide, contrasted with uranyl acetate, and dehydrated in graded concentrations of ethyl alcohol (20, 30, 40, 50, 60, 70, 80, 90, and 100%). Then, transverse thin sections from samples embedded in resin were mounted on grids (300-mesh copper grids; Electron Microscopy Sciences, Hatfield Pennsylvania), followed by treatment with lead citrate. The samples were stained with 1% phosphotungstic acid at pH 7.2 and visualized by TEM. The images were processed at 100,000x and analyzed to determine the cell-wall thickness from an average of 10 cells per bacterial strain. The *S*. *aureus* strain ATCC 25923 and the VISA strain Mu50 ATCC 700699 were used as references.

### Clinical records of pediatric patients

The clinical analysis of the pediatric patients considered the following parameters: source, sex, age, clinical diagnosis total number of days of vancomycin treatment, and clinical outcome (Table 2).

**Table 2:** Clinical characteristics of the 12 VT-MRSA isolates.

Isolate	Source	Sex	Age (Months)	Clinical diagnosis	TVT (days)	Clinical Outcome
179D	Bloodstream	M	168	Chronic renal insufficiency	21	Improvement
428H	Bloodstream	F	4	Gastroschisis	18	Death
74D	Pleural fluid	M	60	Pleural effusion	21	Improvement
148D	Wound	F	NA	NA	NA	NA
645H	Bloodstream	F	NA	NA	NA	NA
440H	Bloodstream	F	60	NA	NA	NA
A17	Bloodstream	F	2	Neonatal sepsis	19	Improvement
163D	Bronchoaspirate	F	156	Iatrogenic gastric perforation	20	Death
480H	Bloodstream	F	2	Choanal atresia and ear anomalies	24	Improvement
250D	Bloodstream	M	84	Hydrocephalus and cardiomyopathy	21	Improvement
737H	Bloodstream	M	144	Chronic renal insufficiency	18	No improvement
817H	Bloodstream	F	2	Intestinal atresia type II	18	Improvement

TVT: Total number of days of vancomycin treatment; NA: Not available

## Results

### Identification of VT-MRSA clinical isolates

No vancomycin resistance was observed among the 100 MRSA isolates examined in the present study. The results of the analysis showed that 12% (12/100) of the MRSA clinical isolates were tolerant of vancomycin (Table 3). Further analysis of the 12 clinical isolates showed three profiles of vancomycin tolerance: 25% (3/12) of the MRSA clinical isolates showed a MBC/MIC ratio of ≥ 32, 50% (6/12) showed a MBC/MIC ratio of ≥ 64 μg/mL, and 25% (3/12) showed a MBC/MIC ratio of ≥ 128 μg/mL (Table 3).

**Table 3:** Phenotypic and genotypic characteristics of the 12 VT-MRSA clinical isolates.

Isolate	MBCμg/mL	MICμg/mL	MBC/MIC	Genes	
				*agr*	*mec*
179D-100406	128	2	64	II	II
428H-240306	128	2	64	II	II
74D-130706	128	2	64	II	II
148D-200706	128	1	128	II	II
645H-110309	32	1	32	II	II
440H-140109	64	2	32	II	II
A-17–180506	128	2	64	II	II
163D-270109	128	2	64	II	II
480H-140109	128	1	128	I	II
250D-26–07–06	128	2	64	I	IVA
737H-200305	32	1	32	I	IVA
817–050406	128	1	128	III	II
ATCC 25923				III	II
Mu50				II	II

### Clinical diagnosis of the 12 patients with nosocomial infections

In the present study, we obtained 100 *S*. *aureus* isolates from pediatric patients from 2005 to 2009, with 12% (12/100) identified as VT-MRSA isolates. The VT-MRSA isolates with clinical relevance were further analyzed to determine the effect of vancomycin on cell wall thickening. Briefly, the patients comprised 66.66% (8/12) females and 33.33% (4/12) males ranging in age from 2 to 168 months. Table 3 provides a description of the total number of days of vancomycin treatment and clinical diagnosis of the 12 patients. In addition, the patients showed significant comorbidities, such as chronic renal insufficiency, gastroschisis, pleural effusion, neonatal sepsis, intestinal atresia type II, choanal atresia and ear anomalies, iatrogenic gastric perforation, hydrocephalus, and cardiomyopathy. Notably, the VT-MRSA clinical isolates were acquired from different sources: 75% (9/12) from the bloodstream and 8.33% (1/12) from pleural fluid, bronchoaspirates, and wounds, respectively (Table 2).

### Antimicrobial susceptibility testing of VT-MRSA clinical isolates

**Fig 1:**
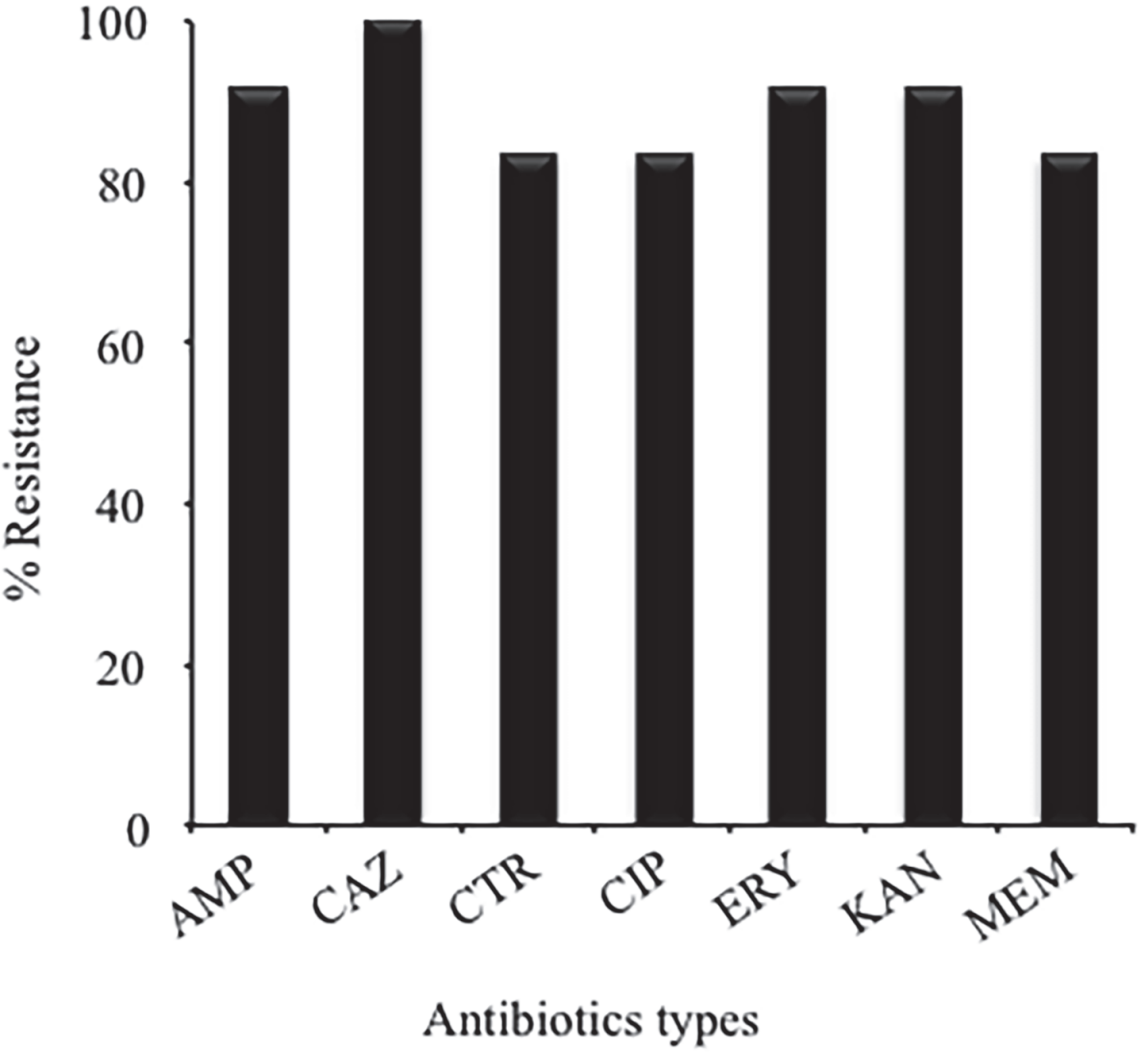
Antibiotic resistance of the VT-MRSA clinical isolates obtained from different pediatric patients. AMP: Ampicillin, CAZ: Ceftazidime, CTR: Ceftriaxone, CIP: Ciprofloxacin, ERY: Erythromycin, KAN: Kanamycin, and MEM: Meropenem.

The VT-MRSA clinical isolates were susceptible to the following antibiotics: vancomycin, gentamycin, rifampicin, and trimethoprim/sulfamethoxazole (Fig. 1). In addition, the MIC values indicated that 100% (12/12) of the VT-MRSA clinical isolates were resistant to ceftazidime, 91.66% (11/12) were resistant to ampicillin, erythromycin, and kanamycin, and 83.33% (10/12) were resistant to ciprofloxacin, ceftriaxone, and meropenem (Fig. 1).

### Identification of the hVISA phenotype among the VT-MRSA clinical isolates

PAP-AUC was used to identify the hVISA phenotype among the VT-MRSA isolates. The results revealed that 41.66% (5/12) of the VT-MRSA isolates showed the hVISA phenotype, growing to 8 mg/mL in the presence of vancomycin concentrations ranging from 0 to 8 mg/mL and with a ratio cutoff value of **≥** 0.9 ± 0.03.

### Distribution of *mec* and *agr* polymorphisms among VT-MRSA clinical isolates

**Fig 2:**
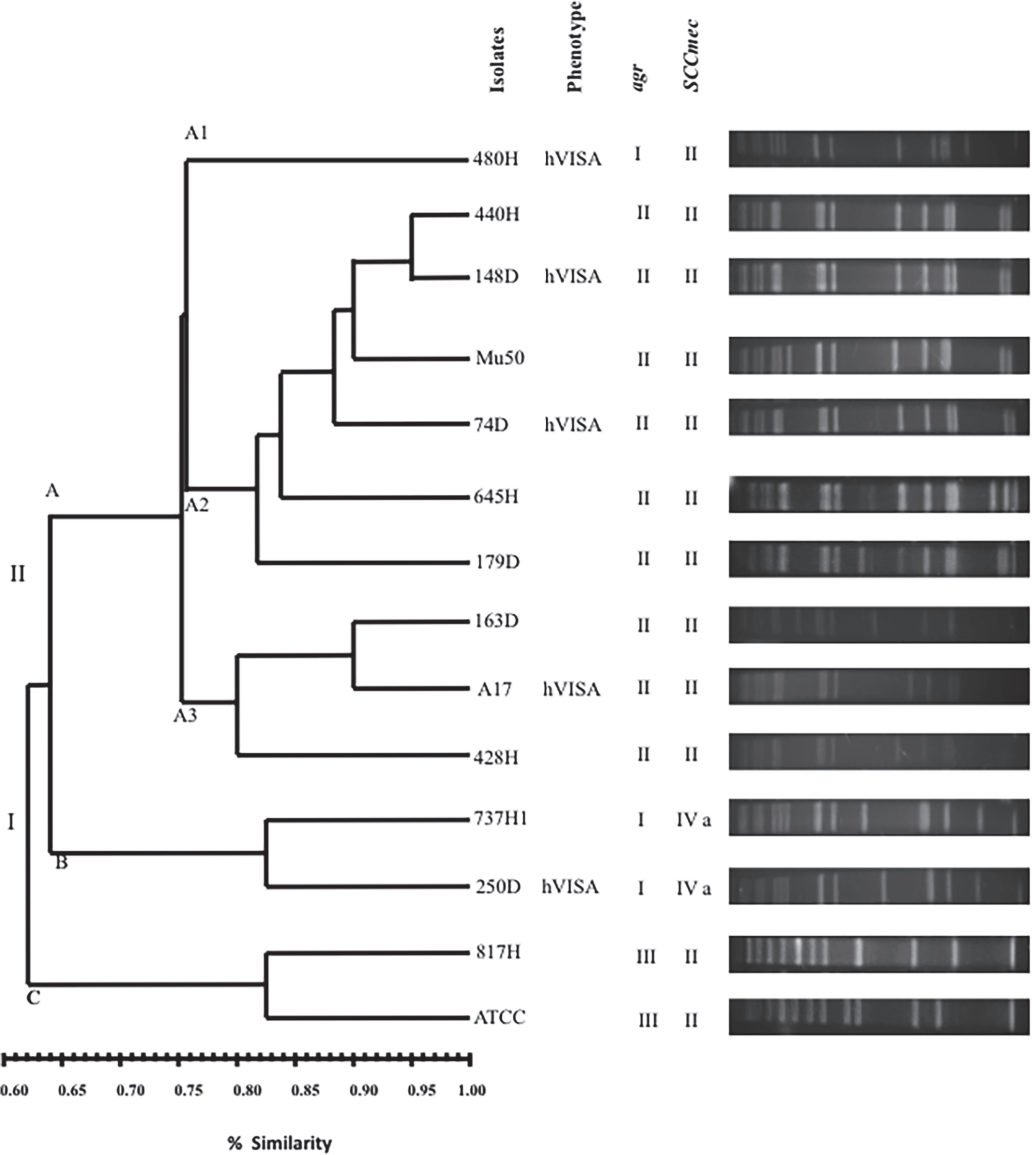
Dendrogram analysis of PFGE results showing the genetic relationships among the PFGE profiles and the presence of the *agr* and SCC*mec* genes among the 12 VT-MRSA clinical isolates.

The multiplex-PCR assays revealed the amplification of a 398-bp product corresponding to the *mec* group II polymorphism in 83.33% (10/12) of the VT-MRSA isolates and an 776-bp product corresponding to the *mec* group IVa polymorphism in 16.6% (2/12) of the VT-MRSA isolates. In addition, a 575-bp product corresponding to the *agr* group II polymorphism was amplified in 66.66% (8/12) of the VT-MRSA isolates, a 441-bp product corresponding to the *agr* group I polymorphism was amplified in 25% (3/12) of the isolates, and a 323-bp product corresponding to the *agr* III group polymorphism was amplified in 8.33% (1/12) of the isolates. The *agr* II/SCC*mec* II polymorphism was observed in 66.66% (8/12) of the MRSA isolates examined. Moreover, the hVISA phenotype identified in the MRSA isolates was associated with three genotypes: *agr*II/*SCCmec*II (60%; 3/5), *agrI/SCCmec*II (20%; 1/5) and *agr*I/*SCCme*IVa (20%; 1/5) (Fig. 2).

### Genetic relationship among 12 VT-MRSA clinical isolates

The PFGE analysis revealed two genetic lineages (I and II) with 5 DNA pulsotypes and 62% similarity, showing patterns comprising 10 to 15 DNA fragments ranging in size from 48.5 to 339.5 kb (Fig. 2). In addition, groups A and B were organized into genetic lineage I, and group C was organized into genetic lineage II. However, nine VT-MRSA clinical isolates sharing greater than 75% homology were identified in the three sub-groups A1, A2, and A3 (Fig. 2). In group A, 88.88% (8/9) of the clinical isolates harbored the *agr* group II polymorphism, and 100% (9/9) of the clinical isolates harbored the *SCCmec* II polymorphism. Interestingly, four hVISA isolates were also identified in this group, whereas the reference strain Mu50 showed to genes both *agr*II and *SCCmec*II. Group B comprised two sub-groups, B1 and B2, each containing a single clinical isolate; both isolates showed genes harboring *agr* group I and *SCCmec* group IVa polymorphisms, and one isolate in sub-group B exhibited the hVISA phenotype. Moreover, group C showed a lower genetic correlation with groups A and B (62% similarity); this group comprised a single clinical isolate and the reference strain ATCC 25923.

### Determination of cell-wall thickening in VT-MRSA clinical isolates

**Fig 3:**
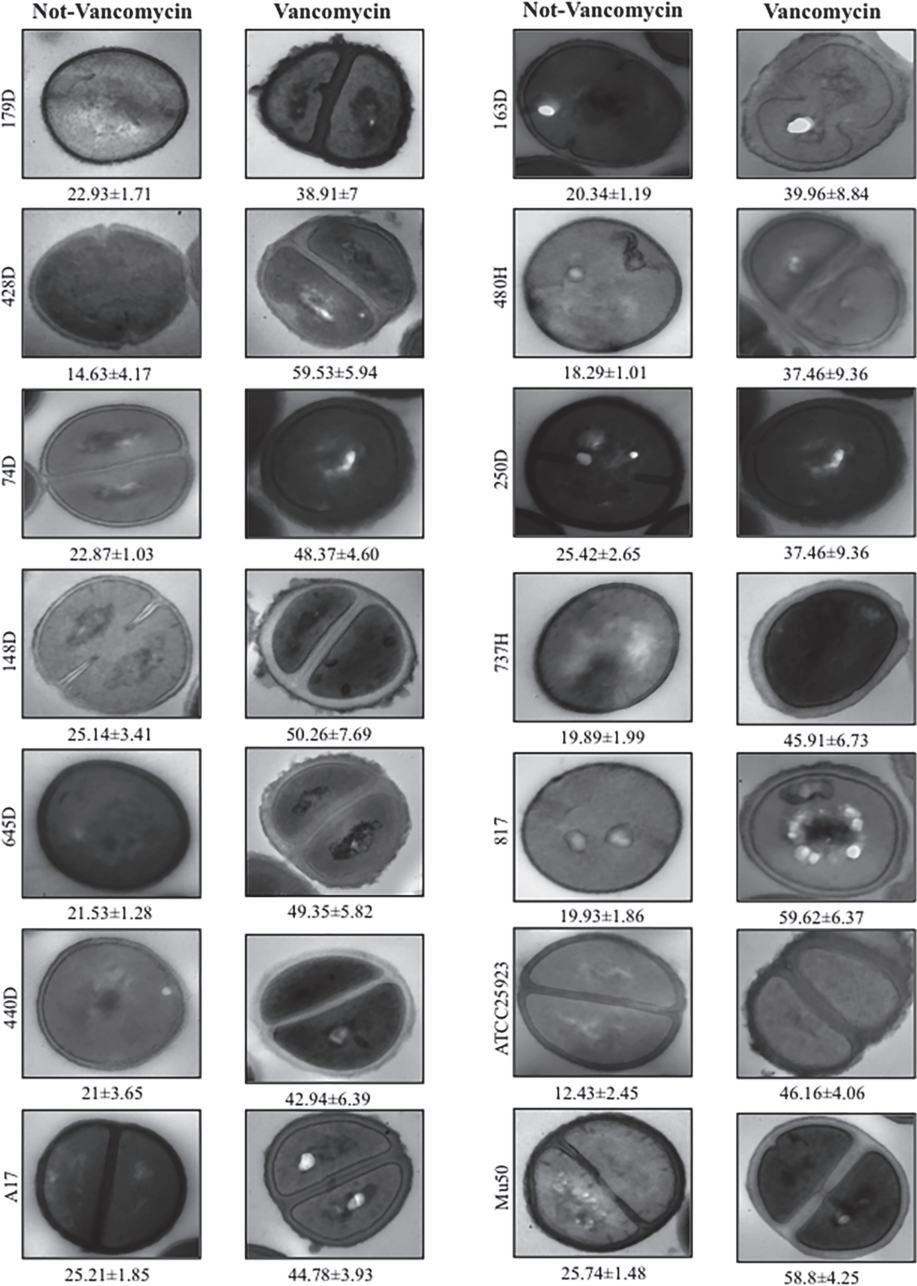
Comparison of the cell wall thickness in the presence and absence of vancomycin among the VT-MRSA clinical isolates after cultivation in BHI broth. (A) Untreated VT-MRSA clinical isolates. (B) VT-MRSA clinical isolates treated with vancomycin at concentrations gradually increased by 1 μg until reaching a final concentration of 20 μg/mL. The micrographs of the thin transverse sections were processed to 100,000x magnification. The values shown under each bar represent the means and SDs of the cell wall thickness in nanometers.

The morphological characteristics of the VT-MRSA clinical isolates are shown in Fig. 3. The 12 VT-MRSA clinical isolates were stimulated with gradual increments of vancomycin, generating cell wall thickening of varying diameters ranging from 37.46 to 59.62 nm (Fig. 3). The unstimulated VT-MRSA isolates showed cell wall diameters ranging from 14.66 to 25.74 nm. Therefore, the average cell wall diameter of the 12 unstimulated (21.46 nm) and stimulated (47.42 nm) VT-MRSA isolates showed a difference of 25.96 nm (Fig. 3 and Table 4). Briefly, the 12 unstimulated VT-MRSA isolates exhibited thinner cell walls than the isolates stimulated with vancomycin.

**Table 4:** Comparison of the cell wall thickness among the 12 VT-MRSA clinical isolates in the presence and absence of vancomycin.

Mean cell wall thickness (nm ± SD^*a*^)				Mean cell wall thickness (nm)			
Vancomycin				Vancomycin			
Isolates	Untreated	Treated	Increase	Isolates	Untreated	Treated	Increase
179D	22.93 ± 1.71	38.91 ± 7.01	15.98	163D	20.34 ± 1.19	39.96 ± 8.84	19.62
428H	14.63 ± 4.17	59.53 ± 5.94	44.9	480H	18.29 ± 1.01	37.46 ± 9.36	19.17
74D	22.87 ± 1.03	48.37 ± 4.60	25.5	250D	25.42 ± 2.65	52.03 ± 8.92	26.61
148D	25.54 ± 1.79	50.26 ± 7.69	24.72	737H	19.89 ± 1.99	45.91 ± 6.73	26.02
645H	21.53 ± 1.28	49.35 ± 5.82	27.82	817H	19.93 ± 1.86	59.62 ± 6.37	39.69
440H	21 ± 3.65	42.94 ± 6.39	21.94	ATCC 25923	12.433 ± 2.45	46.16 ± 4.065	33.73
A17	25.21 ± 1.8	44.78 ± 3.93	19.57	Mu50	25.74 ± 1.48	58.87 ± 4.25	33.13

^*a*^ The morphometric evaluation of the cell wall thickness was performed using the TEM images obtained at 80,000x.

## Discussion

In the present study, 12% of the MRSA clinical isolates were tolerant of vancomycin, with MIC values of 1 and 2 μg/mL; these data are consistent with other reports [6, 37, 38, 39, 40]. Additional studies have shown MRSA strains with vancomycin tolerance rates ranging from 0% to 47% [6, 7, 40, 41, 42, 43]. Our results are consistent with these studies, supporting the relevance of intra-hospital emergent populations such as VT-MRSA clinical isolates associated a nosocomial outbreak. The selection of *S*. *aureus* strains that are tolerant of vancomycin likely reflects exposure to suboptimal concentrations of vancomycin *in vivo*, which could explain the rapid development of hVISA and VISA strains [17, 42]. In the present study, the PAP-AUC analysis revealed 41.66% VT-MRSA strains with the hVISA phenotype. A variable frequency of hVISA strains, ranging from 0 to 50%, has been described [44]. In addition, 29.23% (19/65) of MRSA strains from infective endocarditis patients exhibited the hVISA phenotype [10]. However, among the 100 MRSA strains obtained from Southmead Hospital in the UK, only 7% of them were hVISA strains as detected on gradient plates, and 5% were hVISA strains as detected using the screening method [16]. The hVISA clinical isolates identified in our study are relevant to hospitals due to their ability to acquire vancomycin resistance upon using this antibiotic to treat nosocomial infections.

The *agr* operon has been identified as a significant factor in the development of reduced vancomycin susceptibility and associated treatment failure [6, 24, 25, 33]. In the present study, 66.66% (8/12) of the VT-MRSA clinical isolates exhibited the *agr* group II polymorphism, 25% (3/12) exhibited the *agr* group I polymorphism, and 8.33% (1/12) exhibited the *agr* group III polymorphism. These results are consistent with those of other studies conducted in the United States and Japan, which have reported a high frequency of the *agr* group II polymorphism in the majority of *S*. *aureus* strains [24, 25]. Our data suggest that VT-MRSA clinical isolates exhibiting the *agr* group II polymorphism, confer advantages to the bacteria for survival in vancomycin-treated patients in a hospital environment.

The dendrogram analysis using PFGE showed that 88.88% (8/9) of the VT-MRSA isolates exhibiting *agr* group II polymorphisms and 11.11% (1/9) of the isolates exhibiting *agr* group I polymorphisms were clustered in group A. Therefore, 66.66% (8/12) of the VT-MRSA clinical isolates displayed a profile of highly related (80%) clustering in group A, whereas 16.66% (2/12) of the VT-MRSA clinical isolates were clustered in group B, with the remaining 8.33% (1/12) clustered in group C. The prevalence of the *agr* group II polymorphism observed in the present study is consistent with previous studies showing that *agr* group II isolates are the most prevalent among clinical MRSA strains [45]. Conversely, 100% (9/9) of VT-MRSA clinical isolates displayed a *SCCmec* group II polymorphism, and consistent with the results of the PFGE analysis, these bacteria were clustered in group A. The presence of both polymorphisms (*agr* group II and *SCCmec* group II) was observed in 88.88% (8/9) of the VT-MRSA clinical isolates from the pediatric patients at HIMFG. These data indicate that both polymorphisms identified in the population of VT-MRSA clinical isolates are associated with decreased susceptibility to vancomycin. Interestingly, although 83.33% (10/12) of the VT-MRSA isolates examined in the present study showed the *SCCmec* group II polymorphism, previous studies have reported a low frequency of this genotype [46]. The high prevalence of the *SCCmec* group II polymorphism in VT-MRSA isolates found in this study is likely due to their intra-hospital origin. However, other studies have shown SCC*mec* type IV as the most frequent of the SCC*mec* types, consistent with reports in other countries showing a frequency of 86.7%. Therefore, SCC*mec* type IV has been considered one of the most frequent nosocomial SCC*mec* types observed in several countries [46]. Previous studies have also indicated that the VISA and hVISA clinical isolates from diverse geographic origins with several point mutations show the *agr* group II polymorphism; however, significant diversity in the *agr* polymorphism has been observed in hVISA strains [25, 30, 47]. The increased heteroresistance to glycopeptides and the attenuated bactericidal activity at clinically relevant vancomycin concentrations have been associated with isogenic mutations in the *agr* group II polymorphism [24]. The *agr* type II polymorphism has been strongly associated with vancomycin treatment failures in MRSA bacteremia, reduced bacterial killing due to reduced autolysis, and decreased *agr* function in biofilm formation [24, 39].

Cell wall synthesis is a crucial step during the growth and division of bacteria and represents an important target for antibiotics, such as penicillin, vancomycin, and teicoplanin [48]. The use of vancomycin has led to the emergence of hVISA and VISA strains exhibiting cell wall thickness and reduced autolysis [48, 49]. However, the cell wall thickness in vancomycin-tolerant MRSA isolates has not previously been evaluated. The results obtained in the present study suggest that vancomycin induced cell wall thickening in the 12 VT-MRSA isolates examined. The analyzed images of all isolates showed that the average cell wall thickness was 22 nm for untreated VT-MRSA and 46 nm for isolates treated with vancomycin. Furthermore, an average increase in cell wall thickness of 24 nm (105.55%) was observed in the VT-MRSA isolates. A similar increase in cell wall thickness was observed for the vancomycin-sensitive *S*. *aureus* strain ATCC 25923 and the VISA strain Mu50. In addition, cell wall thickening has been described in gentamicin- and macrolide-resistant *S*. *aureus* clinical strains [50, 51]. The data presented herein strongly indicated that the thickened cell wall in VT-MRSA clinical isolates with *agr* type II and *SCCmec* group II polymorphisms is associated with an adaptive resistance to vancomycin, suggesting that this resistance is inducible. Consistently, other studies have shown that *S*. *aureus* strains with acquired resistance could be reverted to the original state. The phenotypic profiles obtained from molecular analyses (*agr*-types, *SCCmec*-types, susceptibility patterns, and clonal grade by PFGE) and structural studies through TEM (cell wall thickness) on the 12 VT-MRSA isolates did not show a relationship with the clinical data of each pediatric patient.

In conclusion, the results of the present study strongly suggest that cell wall thickness is a major feature of VT-MRSA clinical isolates, conferring resistance at concentrations above the MBC. These findings suggest that the adaptive resistance is inducible and are consistent with other studies suggesting that this adaptive resistance could be reverted to the original state. In addition, the *agr* type II and *SCCmec* group II polymorphisms might also positively contribute to vancomycin tolerance through an increase in the high-affinity trapping and cloning of the mesh in the outer layer of peptidoglycan. Combined with preexisting knowledge, these results suggest that these pathogenic attributes mediate cell wall thickening in VT-MRSA clinical isolates.
